# From Clinical Scenarios to the Management of Lower Urinary Tract Symptoms in Children: A Focus for the General Pediatrician

**DOI:** 10.3390/healthcare11091285

**Published:** 2023-04-30

**Authors:** Pier Luigi Palma, Pierluigi Marzuillo, Anna Di Sessa, Stefano Guarino, Daniela Capalbo, Maria Maddalena Marrapodi, Giulia Buccella, Sabrina Cameli, Emanuele Miraglia del Giudice, Marco Torella, Nicola Colacurci, Carlo Capristo

**Affiliations:** Department of Woman, Child and General and Specialized Surgery, University of Campania “Luigi Vanvitelli”, 80128 Naples, Italy

**Keywords:** overactive bladder, voiding dysfunctions, urinary incontinence, pediatric urogynaecology

## Abstract

Lower urinary tract symptoms (LUTS) are a relevant problem in the pediatric population, having a very high prevalence. Diurnal incontinence and nocturnal enuresis are surely the most frequent symptoms, presenting, respectively, in up to 30% of school-age children and up to 10% of children between 6 and 7 years. Stypsis is the most common comorbidity, and it must be considered in the management of LUTS; indeed, the treatment of constipation is curative in most cases for both incontinence and enuresis. The presence or absence of diurnal symptoms in nocturnal enuresis and urgency in diurnal incontinence helps in the differential diagnosis. Urotherapy is always the first-line treatment, while oxybutynin and desmopressin (where appropriate) may help if the first-line treatment is unsuccessful. It is essential to identify conditions that are potentially dangerous for kidney and urinary tract well-being, for which LUTS can be the first manifestation. Starting from a series of clinical scenarios, we will underline the diagnostic clues behind LUTS in children and we will summarize clinical and surgical approaches for the proper management of these conditions.

## 1. Introduction

Symptoms deriving from the lower urinary tract are grouped under the umbrella term “lower urinary tract symptoms” (LUTS). LUTS are classified by the International Children’s Continence Society (ICCS) according to their relationship with the voiding or storage phase of the bladder cycle or a combination of both in changing severity. Mainly, the conditions are classified into either overactive bladder (OAB) or dysfunctional voiding. Storage symptoms are represented by increased (≥8 times/day) or decreased (≤3 times/day) number of diurnal voiding, daytime continuous or intermittent incontinence, enuresis, urgency, and nocturia. Voiding symptoms are classified as hesitancy, straining, weak stream, and intermittency [1].

LUTS are very frequent in the pediatric population, with daytime incontinence, defined as an uncontrollable leakage of urine, present in up to 30% of school-age children. Holding manoeuvres and urgency, which is a sudden and unexpected experience of an immediate need to void, are also highly prevalent [2]. Common holding manoeuvres are crossing legs, standing on tiptoe, or squatting with the heel pressed into the perineum. Enuresis, defined as intermittent incontinence while sleeping, has a prevalence of 10% in children aged between 6 and 7 years. It is subdivided into monosymptomatic (MNE), without daytime symptoms, and non-monosymptomatic enuresis (NMNE), with the presence of daytime symptoms usually associated with bladder dysfunction and less often with urinary anomalies [3]. Generally, LUTS are more frequent in girls aged between 6 and 8 years [2]. Nocturia is defined as when the child must wake during the night to void and is more frequent among school children [4].

Comorbidities are a relevant problem in these patients. ICCS suggested a list of comorbidities such as constipation, encopresis, urinary tract infection (UTI), vesicoureteral reflux (VUR), and neuropsychiatric conditions (attention deficit hyperactivity disorder, oppositional defiant disorder, learning disabilities, and disorders of sleep) that must be considered when studying LUTS properly. Stypsis is surely the commonest and most important comorbidity: up to 30% of children affected by stypsis have diurnal urinary incontinence and/or enuresis. On the other hand, proper stypsis management alone can resolve 90% of diurnal urinary incontinence cases and 60% of enuresis cases [5]. Holding manoeuvres, decreased voiding frequency, and urgency are associated with the presence of constipation [6]. The compression of the rectum on the bladder wall and the chronic external anal sphincter contraction are responsible for detrusor hyperactivity with frequent and forced contraction of the pelvic floor and anal sphincter itself, which, in turn, could “feed” constipation. This condition is known as bladder and bowel dysfunction (BBD) [7]. Moreover, children with bladder dysfunction are at risk of VUR as a consequence of urine retention in the bladder, with an increased risk of UTI and upper urinary tract damage [8].

LUTS diagnosis is not always easy to reach and so the treatment could be delayed. Firstly, LUTS are often underestimated by primary care paediatricians who tend to wait for a spontaneous resolution of the condition [3]. Secondly, the symptoms themselves are not always reported clearly by the child or his/her caregiver [1]. Voiding frequency is also hard to report precisely to caregivers until they have a chance to assess it with a complete bladder diary. School-age children may have a decreased voiding frequency related to poor hygienic conditions at school, and they could unconsciously suppress the needing of voiding through holding manoeuvres [2]. These problems may not help clinicians in taking an accurate anamnesis, which needs a lot of time to be well studied.

Starting from a series of “illustrative” case reports, we will underline the diagnostic issues and the diagnostic clues behind LUTS in children to help paediatricians in orienting toward the differential diagnosis and the proper management of these conditions.

## 2. Clinical Scenarios

### 2.1. Clinical Scenario 1

An 8-year-old girl was referred to our outpatient clinic for urgency, urge incontinence, and increased voiding frequency (12–14 times/day) in the last year. Before that, no urinary symptoms were described. Nocturnal and diurnal continence was attained at 2.5 years old and no UTI was reported by the mother. No malformations at the external genitalia examination were found. Urine analysis was normal. At the kidney and urinary tract ultrasound (KUS), both kidneys and bladder were normal and no post-void residual volume was found. The uroflowmetry revealed a tower-shaped curve. This clinical picture was suggestive of OAB. Standard urotherapy was the first line of treatment and an anticholinergic agent (oxybutynin) helped in the later management.

### 2.2. Clinical Scenario 2

A 7-year-old boy came to our outpatient clinic with increased daytime voiding frequency (more than 15–20 times) presenting in the last 5 months. No urinary urgency, nocturia, daytime incontinence, or nocturnal enuresis were reported. Stypsis was absent and urinalysis was normal. The clinical examination was unremarkable. The bladder voiding diary revealed 37 micturitions/day, with a volume void of each micturition between 10 and 40 mL. Based on this clinical presentation, the diagnosis of extraordinary daytime-only urinary frequency (EDOUF) was made. We reassured the parents about a benign prognosis and spontaneous resolution [9].

### 2.3. Clinical Scenario 3

A 4-year-old girl was observed for diurnal and nocturnal incontinence without urgency. The urinalysis was normal. The physical examination of external genitalia revealed a bifid clitoris, hypoplasia of minora labia, and patulous urethra due to a dorsal urethral defect. The diagnosis of female epispadias was found. Cystography was normal and the surgical correction of the defects determined the disappearance of the urinary incontinence [10]. At the postsurgical follow-up, no symptoms were reported.

### 2.4. Clinical Scenario 4

A 6-year-old girl presented with a history of daytime incontinence with increased daytime voiding frequency and enuresis. She also reported recurrent febrile UTIs since she was 18 months old. KUS showed bilateral hydronephrosis, with an anteroposterior diameter of the right pelvis of 21 millimetres and the left pelvis of 24 millimetres. The bladder wall was irregular and 6 millimetres thick (bladder fully filled). Moreover, she reported being styptic since she was 2 years old and recurrent nonfebrile UTIs in the last 6 months (about 3 episodes). Uroflowmetry revealed that a “staccato” pattern was present. An antibiotics prophylaxis with amoxicillin and a bowel regulation strategy (stool softeners plus lifestyle modification) were started. The girl was re-evaluated after 3 months, with normalizations of the symptoms, KUS, and uroflowmetry. In this case, the diagnosis was BBD, and all of the symptoms disappeared with proper urotherapy and stypsis management.

### 2.5. Clinical Scenario 5

An 8-year-old girl was observed for persistent urinary incontinence without urgency. She reached continence at 2.5 years old. At the physical examination, the genitalia were normal, but urine dripping was seen during the ostium vaginalis exploration. In the suspicion of an ectopic ureter, a KUS was performed, and a right kidney duplication with a megaureter of 8 mm of the upper pole was found. The Tc-99m MAG3 kidney scintigraphy revealed absent function (6%) of the right upper pole. In addition, we made a methylene blue test consisting of filling the vesical bladder with blue-coloured NaCl 0.9% by vesical catheterization. In this test, we found vaginal non-blue urine dropping, confirming that this dropping was derived from an ectopic ureter in the vagina and not from the vesical bladder. During cystoscopy, the ureteral orifice of the ureter of the upper pole was not found. The nephroureterectomy of the upper pole resolved the incontinence of the patient.

## 3. How to Clinically Orientate towards a Correct LUTS Diagnosis

An accurate anamnesis should be taken, focusing on symptoms reported by the child and confirmed by the caregiver. The paediatrician should investigate voiding patterns, frequency, bowel habits, fluid intake, and posturing taken during micturition. Moreover, a medical history of patients and relatives regarding nephro-urological diseases and other problems (diabetes, trauma, psychiatric diseases, psychosocial history, medications, and surgical history) should be considered. Asking for a history of constipation and recurrent UTIs should be the first step in the management of any urinary symptoms.

Physical examination is essential and sometimes enough to make a diagnosis. Any anomalies should be noted, including ear anomalies, which can be associated with urinary tract malformations [11]. Abdomen palpation could help in assessing stypsis by the presence of stool in the left iliac fossa. Most important is the evaluation of the genitalia in both males and females since continuous urinary incontinence without urgency and nocturnal enuresis could be caused by both hypospadias and epispadias [10,12]. Moreover, if genitalia is normal with urine dripping during an inspection, an ectopic ureter could be suspected, especially in the case of a duplex kidney without megaureter (or rarely with megaureter) of one of the two ureters draining that kidney [13]. In this case, a methylene blue test could be useful to confirm the clinical suspicion (please see clinical scenario 5). Another clinical entity to exclude is intravaginal micturition, which is frequently found in girls with overweight or obesity [14]. In this case, it could be useful to suggest micturition with a proper voiding position—spreading legs and voiding with feet supported—in order to prevent the intravaginal reflux of urines [14].

A urine analysis test is mandatory in excluding both mellitus (glycosuria), diabetes insipidus (low urinary density), and UTI (leukocyturia and/or nitrituria).

At first evaluation, it should always be asked that a bladder diary be completed by the caretaker or patient. ICCS recommends at least a 48 h daytime frequency and volume chart to well evaluate LUTS [7]. However, it depends on patients’ compliance, and it is very subjective; therefore LUTS could be underestimated, and a 16% rate of false negatives for frequency has been seen [15]. Moreover, a bladder diary focusing on wet and dry night frequency could help. Additionally, nocturnal polyuria can be detected in enuretic children by collecting the urine volume produced during the night. To correctly measure night urinal volume, parents should sum the difference between wet diapers in the morning and dry diapers with the volume of the first micturition in the morning [16]. A urine production of >130% of the expected bladder capacity (EBC) is considered pathological [17].

KUS is a noninvasive and useful investigation tool that helps physicians in cases of kidney abnormalities, such as urinary tract dilatations or kidney duplications. Bladder thickness in healthy children should not overtake 3 and 6 millimetres in a full and empty bladder, respectively [18]. Bladder ultrasound is supportive in the diagnosis of bladder outlet obstruction (BOO) in males with increased bladder thickness. The latter could be present in OAB as well [19]. Residual postvoid volume should be assessed after a physiologic bladder filling, and a zero volume should be found in healthy children, while more than 20 mL on repeated measurements indicates voiding dysfunction [1]. Moreover, ultrasounds could be helpful in chronic stypsis diagnosis, since styptic children may have an increased rectum diameter (>3 cm) and a bladder dislocation [20]. Literature data show good reliability of urinary bladder ultrasound in children as far as bladder volume measurement is concerned. Given the variability in bladder wall thickness, a standardized methodology is desirable to increase its reliability [21].

Uroflowmetry is functional for a correct diagnosis. It is considered a noninvasive urodynamic test. Therefore, it is useful in approaching children with urinary symptoms. It consists in letting a toilet-trained child void their bladder into a toilet connected to a sensor that measures urine flow, and it is always associated with a residual postvoid volume measurement at ultrasonography. All of the adaptions possible for children should be taken to let them void their bladder in a position that is normal for them [22]. Uroflow is best performed when the bladder is at 50% of EBC [30 + (age in years × 30) up to age 12 years, after which it is considered 390 mL [23]. ICCS describes five patterns: a “bell-shaped” curve in normal voiding, a “tower-shaped” curve when an explosive detrusor contraction occurs, a “plateau-shaped” curve when there is an organic outlet tract obstruction, a “staccato” curve when there is sphincter overactivity during voiding, and “interrupted” curve when abdominal muscles are used for an acontractile detrusor [1]. Uroflowmetry has a diagnostic value and it could be used to screen children who need a more invasive test, such as cystometry [24]. Both cystometric and uroflowmetric techniques can be associated with pelvic floor electromyography (EMG), which assesses the degree of urethral contraction and relaxation.

Finally, cystometry is surely the most definitive test to diagnose voiding dysfunction and outlet obstruction. It allows paediatricians to measure bladder compliance during the filling phase, calculated by dividing the volume change by the change in detrusor pressure. Both transurethral and suprapubic routes can be used. In any case, cystometry is not mandatory, and most cases are diagnosed with uroflowmetry and KUS association. Indeed, cystometry is associated with adverse effects, e.g., UTI, macroscopic haematuria, and urinary retention in up to 20% of cases [25].

## 4. Therapeutic Approach

Generally, after the treatment of a possible underlying UTI, the first-line approach for any patient with LUTS is standard urotherapy. It is defined as a conservative-based therapy and treatment for LUTS that rehabilitates the low urinary tract [26]. It consists of giving patients and parents advice regarding the correct behaviour to partially or totally resolve the dysfunction. This includes information and demystification about pathology, lifestyle recommendations (increase daytime fluid intake, voiding regularly by day, and voiding before bed), and instructions and behavioural modifications to achieve optimal bladder and bowel habits (the child should relax pelvic floor muscles and sphincter during voiding) [26]. Moreover, if more than two nonfebrile UTIs occur in 6 months, or more than four in a year are reported, antibiotic prophylaxis and adequate hydration are recommended.

In the case that standard urotherapy is unsuccessful, a specific urotherapy is the method of choice. An example is “biofeedback”, consisting in letting a child gain greater awareness of the pelvic floor and sphincter muscles by using external instruments [26]. Up to 80% of patients with a “dysfunctional voiding” diagnosis (defined by ICCS as a child who habitually contracts the urethral sphincter during voiding) could have improvement marked by a reduction in incontinence and UTI frequency using biofeedback [27]. Visual feedback of the uroflow curve and teaching perineal muscle identification by EMG electrodes are the best approaches [27]. Moreover, in the case of standard urotherapy and biofeedback failure, a clean intermittent self-catheterization may be helpful in children with high volumes of post-void residual fluid [28].

A postponing micturition exercise, as in clinical scenario 2, could be used in patients with EDUOF diagnosis for which effectiveness in 98.1% of patients who improved or normalized their voiding frequency has been shown [9]. However, we only recommend the postponing micturition exercise when the EDOUF affects the normal activities of either the children or parents. This recommendation had the scope to permit—for the months in which the EDOUF is more disturbing—normal daily activities for both children and parents. Moreover, with this suggestion, we did not aim to use retention control training, which is not recommended for any bladder dysfunction in childhood, but to give suggestions regarding correct micturition habits in order to establish a normal number of daily micturitions and to re-establish a cycle of routine bladder filling and emptying [29].

In patients with MNE, standard urotherapy is more effective than spontaneous cure by a rate of 15% per year [30]. Robson et al. saw a 50% reduction in bedwetting with urotherapy in 40% of the population, while 22% of children did not need any other treatments [31]. Moreover, ICCS recommend starting with urotherapy even in patients with NMNE [1].

MNE should be treated with a combination of urotherapy and desmopressin or alarm therapy. The latter consists in putting a sensor in the night clothes, which gives an auditory signal that wakes the child when it gets wet. The alarm should be preferred in children with a bladder capacity smaller than expected, who are likely to be resistant to desmopressin. It should be worn every night, and treatment should be continued until the patient is dry for 14 consecutive days or 2–3 months [32]. This approach is very family compliance-related since it could be very disturbing for parents and the child himself, and, therefore, it may not be accepted. In this case, treatment with desmopressin is preferable, available as a fast-melting oral lyophilization at a dosage of 120 µg increasing to 240 µg in the case of absent or only a partial response to the previous dosage [33]. Desmopressin is a vasopressin analogue, which, with its antidiuretic effect, concentrates urine, reducing the total volume of urine in the bladder. It is indicated in the case of nocturnal polyuria with a normal bladder capacity or when alarm therapy has failed [32]. Desmopressin is well tolerated; however, rarely, patients may, consequently, develop hyponatremia due to water intoxication, with symptoms including nausea, vomiting, headache, cerebral oedema, and convulsions (especially for the intranasal administration) [31]. To avoid side effects and to optimize the results, patients should reduce the fluid intake from 1 h before to 8 h after the desmopressin consumption. The response rate to desmopressin is higher in patients with frequently recurring nocturnal polyuria [17]. In the case of a partial response (50–99% of wet nights) or the absence (<50% of wet nights) of a response to desmopressin alone, combination therapy with desmopressin plus oxybutynin can increase the response rate [34]. Alarm therapy and desmopressin treatment have similar efficacy in treating MNE [35].

In the case of OAB, urotherapy is still the first choice [36]. Anticholinergic agents, such as oxybutynin and tolterodine, should be used in the case of urgency persistence after standard urotherapy. They have antimuscarinic effects, blocking M_2_ and M_3_ receptors, which are predominantly expressed by bladder smooth muscle. Oxybutynin, at a dosage range from 0.3 to 0.5 mg/kg/day with a top dose of 10 mg [34], is preferred with its superior cost-effectiveness, and both immediate- and extended-release preparations can be used. Adverse effects are present in up to 30% of cases and include dry mouth, dizziness, constipation, somnolence, nausea, blurred vision, urinary hesitation, urinary retention, and dyspepsia, with less incidence of the extended-release preparations. Tolterodine is preferred in the case of serious or disturbing adverse effects to oxybutynin since it is more selective for the bladder and thus there are fewer adverse effects and greater efficacy [37]. Moreover, in the case of bladder-neck dysfunction, an α-blocker treatment may be useful in reducing the voiding dysfunction [38].

In case of unresponsiveness, transcutaneous electrical nerve stimulation (TENS) could be considered. It consists of inhibiting the presynaptic afferent neurons carrying impulses from the bladder by stimulating the nerves of the peripheral segmental dermatome [39]. Finally, an intravesical injection of botulinum toxin A may help to reach complete continence, despite a few adverse effects reported [38].

In conclusion, BBD and recurrent cystitis should be always excluded and eventually treated, as in scenario 4. In the case of the persistence of LUTS, a diagnosis revaluation with deeper diagnostic tools is required.

## 5. When a Surgical Approach Is Needed

Clinical scenarios 3 and 5 represent examples in which a clinical approach is not enough. In the case of female epispadias, an accurate genitalia examination is sufficient, and early surgical intervention is fundamental to let the patient achieve continence and preserve the urinary tract. 

An ectopic ureter is a more complex condition, and the diagnosis could not be as immediate as epispadias. The term “ectopic ureter” describes a ureter that inserts at or distal to the bladder neck, and it is more frequent in females [40]. Frequently, the ectopic ureter drains the upper pole of a duplex kidney, and, while rare, it drains a hypoplastic or dysplastic kidney [40]. In 35% of cases, the insertion is in the urethra, in 34% of cases, it is in the vestibule, and in 25% of cases, it is in the vagina [13]. Clinically, patients describe not being able to reach dryness during the daytime or night, with persistent continuous incontinence. KUS may be able to detect a duplex kidney, while DMSA scintigraphy is useful in detecting poorly functioning renal tissue. A vaginoscopy and a cystoscopy may help in the identification of the ectopic ureter orifice [40]. Patients may undergo many diagnostic procedures and the diagnosis may take a long time with a 5.7-year delay from symptoms presentation to diagnosis [40]. Despite its rarity, an ectopic ureter may be found in male children as well, where it drains a hypoplastic kidney more frequently [41].

A surgical approach is needed in the case of posterior urethral valves (PUV), which are the most common congenital cause of lower urinary tract obstruction. PUV is usually diagnosed during routine prenatal ultrasounds with the detection of hydronephrosis, a thick-walled bladder, and a keyhole sign in the bladder neck of males (dilated prostatic urethra) [42]. In the case of a misdiagnosis, mild PUV could present in older children with recurrent UTIs and LUTS such as delayed voiding and weak stream. Moreover, PUV may be identified in children with renal failure, proteinuria, or through hydronephrosis studies [42]. A voiding cystourethrography (VCUG) is the gold standard imaging for diagnosis [43], and renal scintigraphy is needed to evaluate both kidneys’ differential function. Catheter drainage (when appropriate) and plan endoscopic valve ablation, if the child is stable, seem to be the best approach [42].

## 6. Potential Long-Term Impact on Health and Quality of Life of Children

Proper management, with rapid diagnosis and treatment, is critical in children with LUTS because of the negative influence that LUTS can have on the quality of life. Both enuresis and daytime incontinence affect children and their families socially, behaviourally, and emotionally, with a risk of social isolation, peer conflict, and classroom challenges. Moreover, LUTS affect the individual in terms of development, self-esteem, and performance, as well as the relationship within the family, causing a high level of stress. Rangel et al. concluded that enuretic children have a low quality of life compared to non-enuretic children, without any differences in gender or age [44].

Additionally, it is known that children with both OABs have a prevalence of VUR between 14% and 47%. On the same line, the treatment of overactivity has a positive effect on the resolution and downgrading of VUR [8].

Moreover, the chronic obstruction caused by PUV can lead to hypertrophy of the bladder wall and detrusor muscle, with changes in bladder compliance. The consequent increment in intravesical pressure can cause VUR, and, thus, recurrent infection and progressively impaired renal function [42].

Finally, dysfunctional voiding can present indistinguishably from the classical neurogenic bladder, and such patients may encounter bilateral hydronephrosis and renal failure [45]. Both conditions are associated, as well as PUV, with a bladder pressure increase, with a risk of VUR, hydronephrosis, recurrent UTIs, and end-stage renal insufficiency.

## 7. Conclusions

**Figure 1:**
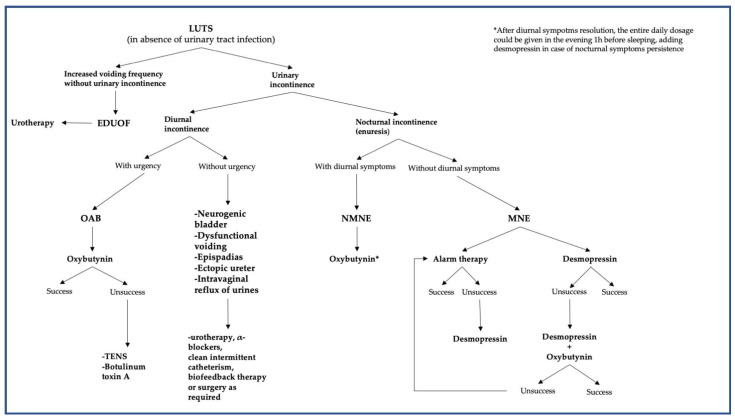
The diagnostic clues and the proper therapeutic approach for LUTS. Abbreviations: EDOUF, extraordinary daytime-only urinary frequency; MNE, monosymptomatic nocturnal enuresis; NMNE, non-monosymptomatic nocturnal enuresis; OAB, overactive bladder; and TENS, transcutaneous electrical nerve stimulation.

LUTS are very common conditions in the pediatric population. They are usually underestimated and their diagnosis is not always immediate, with a frequent need to reevaluate patients. Many diagnostic tools are available to help clinicians orientate through clinical issues of LUTS. A clinical approach starting with urotherapy is usually sufficient for the management, while a few conditions need a surgical approach. In brief, urinary incontinence should be divided into nocturnal and diurnal incontinence. In the former, the co-presence of diurnal symptoms makes for a diagnosis of NMNE, while their absence makes for a diagnosis of MNE. In the case of diurnal incontinence, the presence or absence of urgency should be considered when making a differential diagnosis between OAB and other conditions that could require surgical intervention, such as an ectopic ureter. Medications, such as oxybutynin (in the case of NMNE or OAB) and desmopressin (MNE), are mostly used in the case of unsuccessful urotherapy. Figure 1 synthetizes the diagnostic clues and the proper therapeutic approach for LUTS. LUTS should never be underestimated since they can hide pathologies that could put a child’s health in trouble.

## Data Availability

The data presented in this study are available upon request from the corresponding author.
